# Fine Scale Phylogeography of Urban Western European Hedgehog *Erinaceus europaeus* in South‐East England

**DOI:** 10.1002/ece3.71729

**Published:** 2025-07-17

**Authors:** Jessica Turner, Chris Carbone, Becki Lawson, Katharina Seilern‐Macpherson, Chris Faulkes

**Affiliations:** ^1^ Institute of Zoology, Zoological Society of London London UK; ^2^ School of Biological and Behavioural Sciences Queen Mary University of London London UK

**Keywords:** *Erinaceus europaeus*, mammal, mitochondrial diversification, phylogeography, Western European hedgehog

## Abstract

Preserving genetic diversity within species and populations is an important conservation goal, yet observed genetic structure can be influenced by both contemporary and historical processes. Here, we investigate the phylogeography of the western European hedgehog ( *Erinaceus europaeus* ) in Greater London, east and south‐east England, using DNA sequence analysis of a section of the mitochondrial cytochrome b gene and control region. We find seven haplotypes within the study region, of which six are novel, indicating higher diversity than previously identified in the UK. Comparison with published haplotypes from mainland Europe identified low sequence divergence from those found in France and Jersey. Most haplotypes were widespread within south‐east England, in contrast to strong spatial clustering of haplotypes seen at broader scales across Europe. However, local haplotype diversity varied within the study region, with some evidence supporting the view that genetic isolation has led to an absence of haplotypes in urban sites in central London. Broader UK‐wide sampling is recommended to ascertain whether additional haplotype lineages and geographic structuring exist at larger spatial scales in the UK. These findings may have important considerations for future hedgehog conservation efforts.

## Graphical abstract

Detailed investigation of the phylogeography of western European hedgehogs in Greater London, east and south‐east England using DNA sequence analysis of a section of mitochondrial *cytb* gene and control region identifies higher haplotype diversity than previously found in the UK. Little local‐scale spatial structuring is seen, in contrast to broader scales across mainland Europe, but there is some evidence of potentially low haplotype diversity in isolated urban sites in central London. These findings may have important considerations for future hedgehog conservation efforts.

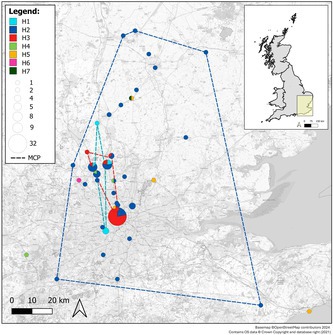

## 1 Introduction

Genetic diversity is a vital component of ecosystem resilience and functioning and is fundamental for species adaptation to their environments (Hoban et al. 2023). Loss of genetic diversity has been linked to a higher risk of population extinction (Frankham 2005), and reduced capacity to respond to environmental changes (Sgrò et al. 2011), therefore maintaining genetic diversity is a global conservation priority.

Both recent and historical processes influence current genetic diversity in populations (Hewitt 1999), and awareness of these impacts can facilitate a more complete understanding of genetic diversity over spatial and temporal scales (Hewitt 1999; Ali 2020). To understand historical demographics and evolutionary origins of populations and species, phylogeography can be used to investigate the geographical distribution of genealogical lineages (Hewitt 2004). In mammals, this approach often utilises the fast‐evolving and nonrecombining mitochondrial DNA to infer matrilineal relationships (Avise et al. 1987).

In Europe, climatic oscillations in the Pleistocene drove substantial cyclical range shifts in temperate species which were restricted to southern refugia during ice ages (Hewitt 1999). After the last glacial maximum (LGM) approximately 19,000 years ago (Clark et al. 2009) species recolonised northwards (Hewitt 1999), but the impacts of long periods of isolation in refugia, varying postglacial colonisation routes, followed by admixture and hybridisation at contact zones, have strongly structured the biogeographic distribution of genetic diversity in many European species (Santucci et al. 1998; Taberlet et al. 1998; Hewitt 1999; Schmitt 2007). However, the role of historical processes on current genetic diversity patterns is not often considered (Dufresnes et al. 2013; Montgomery et al. 2014).

The western European hedgehog ( *Erinaceus europaeus* ) is a small, spiny mammal found across western Europe that has experienced strong declines in much of its range, leading to recent reclassification as Near Threatened on the IUCN Red List (Gazzard and Rasmussen 2024). In the UK, the population has declined severely, particularly in rural environments (Wembridge et al. 2022), and is considered vulnerable to extinction in the Red List for Britain's Mammals (Mathews and Harrower 2020).

The broad‐scale phylogeography of the hedgehog species found in Europe has been well studied (Santucci et al. 1998; Seddon et al. 2001) and forms one of the three paradigms for species recolonisation of Europe put forward by Hewitt (1999). In this, hedgehogs followed a column‐like recolonisation northwards from refugia in the Iberian, Apennine and Balkan refugia (Hewitt 1999) forming three column‐like stripes across Europe, and a fourth in Israel and Türkiye (Hewitt 1999). The western European hedgehog moved northwards from the Iberian and Apennine refugia, and consists of three monophyletic clades, as indicated by mitochondrial sequence analysis (Seddon et al. 2001). These are designated as: E1, which expanded northwards from the Apennine Peninsula into central Europe; E2, which colonised northwards from the Iberian Peninsula along the western edge of Europe; and E3, which is restricted to Sicily (Santucci et al. 1998). More recent analyses have further revealed that the E2 lineage originated from the northeastern region of the Iberian Peninsula, which is characterised by low haplotype diversity and is separated from a higher diversity clade in the northwest of the region by the Ebro River (Sanz et al. 2021).

However, finer scale phylogeographic patterns of the western European hedgehog are not as well understood, and in the UK only limited numbers of samples have been included in previous studies (three in Santucci et al. 1998 from one location; 28 in Seddon et al. 2001 from 10 locations). These suggested a lack of mitochondrial variability, with a single widespread haplotype present across much of Great Britain, which is also found in Ireland, France, Jersey and the Netherlands. However, one location in the south of England also contained two individuals of a second haplotype, which raises the question as to whether further unidentified mitochondrial diversity could be present.

Given the rapid population decline of hedgehogs in the UK (Wembridge et al. 2022), investigation of the local phylogeographic structure of hedgehogs is valuable to investigate genetic diversity and population structuring across the country. In Central Europe, for example, western European hedgehogs were found to have clustered mitochondrial lineages despite homogenous nuclear diversity, suggesting sex‐biased dispersal and female philopatry (Bolfikova and Hulva 2012). Similarly, in Spain, the lack of diversity of western European hedgehog mitochondrial haplotypes north of the Ebro River was interpreted as evidence of the river's role as a semipermeable barrier for hedgehogs, restricting gene flow (Sanz et al. 2021).

Furthermore, urban environments are considered an important habitat for hedgehogs (Wembridge et al. 2022), yet the species is also vulnerable to habitat loss and fragmentation within these landscapes (Turner et al. 2021; Taucher et al. 2020). There is a question over whether urban areas may act as a geographic barrier at the landscape scale, impacting the species phylogeographic structure. One way to investigate this is to look at the presence of geographically localised haplotypes and local differences in haplotype frequencies, which may indicate population substructure within phylogeographic regions (Avise 1992). Knowledge of genetic structuring and relationships among regions can be relevant for management decisions, particularly those which may disrupt genetic structuring such as translocations or reintroductions (Finnegan et al. 2008).

The present study uses detailed sampling to examine western European hedgehog (hereafter referred to as hedgehog, unless otherwise specified) phylogeography and mitochondrial diversity in an urban landscape of Greater London and surrounding counties in the east and south‐east of England. The study aimed to (i) identify mitochondrial diversity present in the study area, (ii) investigate population substructure and gene flow in the region by examining the spatial distribution and diversity of haplotypes and (iii) place these results into the context of broader hedgehog phylogeographic relationships and large‐scale structuring of haplotypes in mainland Europe from a previous study conducted across the species range.

## 2 Methods

### 2.1 Sample Collection

A total of 125 tissue samples from deceased hedgehogs were collected from 2012 to 2022 from Greater London (65) and surrounding counties of Hertfordshire (40), Cambridgeshire (6), Surrey (3), Kent (2), Essex (2), Bedfordshire (1), Suffolk (1), Berkshire (1), and four samples of unknown origin. Samples were provided by the Garden Wildlife Health project (56), a national wildlife disease surveillance scheme, from the −20°C tissue archive housed at ZSL (2012–2020), as well as from rescue centres (38) and were collected opportunistically (e.g., roadkill) by volunteers (31). Samples were initially stored at −80°C for at least 24 h, with the exception of the samples from the Garden Wildlife Health project, before transfer to storage at −20°C. Overall, 125 samples were collected from a mixture of clustered and individual sampling locations. Both the individual coordinates and a more general sampling location were recorded, in which hedgehogs collected from within the same town or within 1 km of each other were considered to be from the same location.

### 2.2 DNA Extraction, Amplification and Sequencing

DNA was extracted using a Qiagen DNeasy Blood and Tissue kit according to the manufacturer's instructions, with the modification that 40 μL of proteinase K was added when extracting DNA from older and more deteriorated samples, where only hair or skin could be obtained (*n* = 26), and overnight lysis of all samples. Where possible, muscle, ear or digit tissue was preferentially used, followed by skin or hair follicles dependent on sample condition. DNA extraction from spine tissue yielded low concentrations (typically < 1 ng/μL) and high levels of impurities when assessed with a Nanodrop One spectrophotometer (Thermo Fisher Scientific) and was therefore not used for PCR amplification reactions.

Hedgehog specific primers were used to amplify a 423 base‐pair region of the cytochrome b (*Cytb*) gene and a 455 base‐pair region of the control region (*CR*). The *Cytb* primers L14724_Hh and H15149 were modified from Irwin et al. (1991) to improve specificity to the hedgehog mitochondrion sequence (Krettek et al. 1995), and the *CR* primers ProL‐He and DLH‐He were from Seddon et al. (2001). The primer sequences and corresponding position of the 5′ primer ends on the hedgehog mitochondrion genome (Krettek et al. 1995) are shown in Table 1. The combination of partial *Cytb* and *CR* regions was selected to enable direct comparison of the haplotypes from this study with previous western European hedgehog haplotypes identified across mainland Europe, including haplotypes previously identified in Great Britain (Seddon et al. 2001).

**TABLE 1:** Table showing the forward and reverse primers used for cytochrome b (*Cytb*) and control region (*CR*) amplification, and their corresponding position in the hedgehog mitochondrial genome (Krettek et al. 1995).

Gene	Primer	Sequence	5′ position
*Cytb*	L14724_Hh	5′CGCGGCCTATGATATGAAAAATCATTGTTG3′	14,131
	H15149	5′GTAGCGCCTCAGAATGATATTTGACCTCA3′	14,584
*CR*	ProL‐He	5′ATACTCCTACCATCAACACCCAAAG3′	15,347
	DLH‐He	5′GTCCTGAAGAAAGAACCAGATGTC3′	15,887

The PCR mix was made up as follows: 10 μL of 5× buffer and 0.5 μL of Taq enzyme (PCR Biosystems Ltd., London, United Kingdom), 2 μL of 10 pmol/μL forward primer, 2 μL of 10 pmol/μL reverse primer, 33.5 μL of ddH_2_O and 2 μL of DNA. Standard *Cytb* PCR conditions were used (Faulkes et al. 1997): 94°C for 3 min, followed by 35 cycles of 94°C for 30 s, 45°C for 45 s and 72°C for 45 s, then 72°C for 5 min and a final hold at 10°C. For *CR*, the same PCR mix was used, but the amplification reaction conditions were optimised to the following: 94°C for 3 min, followed by 35 cycles of 94°C for 30 s, 55°C for 45 s, and 72°C for 45 s, then 72°C for 5 min and held at 10°C.

The PCR products were then visualised on a 1% agarose gel, and successfully amplified sequences were cleaned using either a Monarch PCR & DNA Cleanup Kit, Monarch Exo‐CIP Rapid PCR Cleanup Kit or the Qiagen QIAquick PCR Purification Kit. Purified PCR products were subjected to Sanger sequencing in the forward direction using the according primers (Eurofins Genomics TubeSeq sequencing service; Eurofins Genomics, Ebersberg, Germany). While bidirectional Sanger sequencing is preferred, unidirectional sequencing was used due to cost limitations and was considered sufficient for the short length of the target DNA regions.

## 3 Phylogenetic, Phylogeographic and Genetic Structure Analyses

DNA sequences were aligned manually in Mesquite version 3.70 (Maddison and Maddison 2021), and the sequencing electropherogram inspected using FinchTV version 1.4.0 (Geospiza Inc. 2006) to guide the removal of low‐quality base calls and visually inspect and check sites with substitutions. Primers and ragged ends were trimmed, and the *Cytb* and *CR* sequences concatenated into single haplotypes of 801 bases in length for further analysis.

Haplotypes were identified in ARLEQUIN version 3.5.2.2 (Excoffier and Lischer 2010), including sites with gaps, and diversity indices calculated. These were the number of haplotypes (*h*), absolute and relative frequency of haplotypes, number of polymorphic sites (*S*), nucleotide diversity (*p*), haplotype diversity (*H*_d_) and mean number of pairwise differences (*k*). Tajima's *D* (excluding gaps) and Fu's *F* tests of neutrality were also performed, which infer evidence of past demographic changes such as population expansion. All diversity analyses were also calculated in DnaSP v6 (Rozas et al. 2017), which removes sites with gaps from consideration by default. As a two base‐pair insertion was identified in the *CR* region of our haplotypes, we retained and present the analyses from ARLEQUIN, which uses all sites. The results when gaps are excluded are presented in Table S1 and do not change the inferences.

To investigate haplotype relationships, a haplotype network was constructed in NETWORK v 10.2 (Fluxus‐engineering.com) using the median‐joining network algorithm (Bandelt et al. 1999) and default parameters. Haplotype networks have been highlighted as more appropriate than bifurcating phylogenetic trees for identifying intraspecific haplotype relationships with few mutational differences (Posada and Crandall 2001). In addition, haplotypes were mapped across the study area using QGIS v 3.16 ‘Hannover’ (QGIS Development Team 2020), with pie charts used to represent the proportion of each haplotype in locations with multiple samples.

Spatial structuring in the geographic distribution of haplotypes was investigated by calculating the Minimum Convex Polygon (MCP) area over which haplotypes were found using the ‘adehabitatHR’ package (Calenge 2006) in R Version 4.3 (R Core Development Team 2023). As adehabitat requires at least five points to calculate the MCP, the analysis was only performed for haplotypes occurring at least five times in the data set. Haplotypes occurring less frequently were grouped together to form a single haplotype ‘HX’ representing all ‘other’ haplotypes in the data. This allowed all sample sites to be included in the resampling and MCP calculations for the haplotypes of interest.

Null models of the area over which a haplotype was expected to be found, given its frequency in the data set, were then established using a bootstrapping approach similar to that in Crees et al. (2016). For this, each haplotype was randomly resampled and reassigned to the sampling locations 1000 times, and the MCP calculated for each run. The mean and 95% bootstrap confidence interval of the expected area for each haplotype was then calculated and plotted on a histogram of the null distribution using the ggplot2 package (Wickham 2016), alongside the observed MCP area of the haplotype. If the observed MCP area was smaller than the range of the confidence interval, the haplotype was significantly geographically localised, and if it was greater, it was considered significantly more widespread than expected under a null distribution.

The local diversity of haplotypes was examined by randomly resampling and reassigning haplotypes across the sample locations 1000 times. For each run, the number of haplotypes assigned to each location was recorded. The mean and 95% bootstrap confidence interval for the expected number of haplotypes at each location with at least three samples (*n* = 8 locations) under a null distribution were calculated and plotted onto a histogram alongside the observed number of haplotypes at each site.

To place the findings of this study into the broader European context, haplotypes identified in this study were also compared with those identified in the western European hedgehog in Seddon et al. (2001), the largest study of hedgehog phylogeography across Europe. *Cytb* and *CR* sequences were downloaded from GenBank and combined haplotypes constructed using details given in the original paper (Seddon et al. 2001). These were then aligned to the haplotypes identified in this study. A single sequence of each haplotype was retained for use in the construction of a maximum likelihood (ML) phylogenetic tree in MEGA 11 (Tamura et al. 2021), with 1000 bootstrap replicates, using the HKY + G model which was identified as most appropriate for the data set using model selection in MEGA 11. The 50% consensus tree was retained with nodes appearing in fewer than half of the replicates reduced to polytomies. Bootstrap support labels were only retained for nodes with at least 50% support. A single haplotype was also downloaded for the southern white‐breasted hedgehog ( *E. concolor* ), from the same study, to use as an outgroup to root the tree.

A Bayesian phylogenetic tree was constructed in BEAST v1.10.4 (Suchard et al. 2018) with the same set of sequences described above, using the HKY + G substitution model, strict molecular clock and constant coalescent population size for molecular dating analyses. The tree was calibrated on the UK haplotypes (mean: 13,750 years ago, 95% CI: 10,700–17,200 years ago) using subfossil evidence that hedgehogs had recolonised Europe after the last glacial maximum and were present in the UK by the end of the Pre‐Boreal (Sommer 2007). An MCMC length of 1,000,000 was used with sampling every 1000 iterations. The log file was viewed in Tracer v1.7.2 (Rambaut et al. 2018) to ensure that sufficient burn‐in iterations were used and that ESS exceeded 200 for all priors. A Maximum Clade Credibility Tree was produced in TreeAnnotator v1.10.4, excluding the first 100,000 iterations as burn‐in. The tree was drawn in Figtree v1.4.4 (Rambaut 2018) using 95% highest posterior density (HPD) node intervals to examine divergence times among haplotypes.

Phylogeographic range evolution was explored using Lagrange‐NG (Bettisworth et al. 2023), which uses a dispersal‐extinction‐cladogenesis (DEC) model to infer the most likely ancestral ranges based on a time‐calibrated phylogenetic tree and current range information. In this, the time‐calibrated Bayesian phylogenetic tree was used as input, after pruning to remove the *E. concolor* outgroup and haplotype belonging to the E3 clade, which is restricted to Sicily. A reduced set of seven geographic regions was used to represent the distribution of the data set; these were: Switzerland–Italy, Denmark–Norway–Sweden, Estonia–Russia, Germany–Austria–Poland, France–Netherlands–Jersey, Spain–Portugal and UK. The results were visualised using Lagrange‐NG‐plotter (Bettisworth et al. 2023).

A second median‐joining haplotype network was also constructed in NETWORK v 10.2 containing sequences from this study and mainland Europe. For this, the *E. concolor* outgroup was included to root the haplotype network. As for the phylogenetic tree, only a single sequence was included to represent each haplotype, except for where haplotypes were identical, either due to exclusion of their divergent sites (*n* = 3 haplotypes) or due to being found in both this study and in mainland Europe (*n* = 2 haplotypes). Haplotype networks were drawn in NETWORK Draw and exported for final annotations and colouration in Illustrator (Adobe Inc. 2019), with haplotypes coloured by country of origin in order to identify geographic relationships among the sequences. To compare the local‐scale patterns of geographic structure in our study region to those seen at the broader European scale, *E. europaeus* haplotypes from Seddon et al. (2001) were mapped in QGIS v 3.16 ‘Hannover’ (QGIS Development Team 2020), and MCPs constructed for those haplotypes occurring at least five times. The spatial clustering of haplotypes at the European scale was examined by generating null MCP distributions, as described above, firstly for all western European hedgehog haplotypes from Seddon et al. (2001), and then for clades E1 and E2 separately to account for their different recolonisation histories.

## 4 Results

Both *Cytb* and *CR* were successfully sequenced for 102 samples and concatenated into haplotypes of 801 bases long. Of these, 800 sites were useable with less than 5% missing data. The sequences start at site 14,771 in the hedgehog mitochondrial genome (Krettek et al. 1995). The *Cytb* region was 376 bases long and contained two variable sites, and the control region was 425 bases long with five variable sites, including a two‐base insertion/deletion. Overall, this gave rise to seven haplotypes used in further analyses, shown in Table 2. One haplotype, H2, was identical to E201/01 identified previously in Seddon et al. (2001). Two new *Cytb* haplotypes were identified in H6 and H7, and four new *CR* haplotypes in H1, H3, H4 and H5. The new nucleotide sequences are deposited in the GenBank database under the accession numbers PQ772616‐PQ772621. There was low divergence between haplotypes, with between one and four nucleotide differences between them.

**TABLE 2:** Variable sites in the haplotypes identified in this study.

Haplotype	Site						
	14,910	15,079	15,158	15,159	15,205	15,222	15,560
H1	C	A	—	—	C	A	C
H2	*	*	*	*	*	**G**	*
H3	*	*	A	T	*	*	*
H4	*	*	*	*	*	G	T
H5	*	*	*	*	T	G	*
H6	*	G	A	T	*	*	*
H7	T	*	*	*	*	G	*

*Note:* Nucleotides are shown for haplotype H1 in the top row, and sites matching H1 in subsequent haplotypes are indicated with *, and—indicate gaps. Site number refers to the position in the hedgehog mitochondrion genome (Krettek et al. 1995). H2 is shown in bold as it was previously identified in Seddon et al. (2001) as E201/01. All other haplotypes are newly identified.

### 4.1 Genetic Diversity

The results of the diversity indices and tests of neutrality performed in ARLEQUIN are shown in Table 3, and the frequencies of each haplotype are shown in Table 4. Overall diversity was low, with a nucleotide diversity of 0.00195 (± 0.0013) and an average 1.55 (± 0.939) pairwise differences between haplotypes. Neither test of neutrality was significantly different from zero, indicating a stable demographic history within the study area.

**TABLE 3:** Diversity indices and neutrality test statistics calculated in ARLEQUIN. Mean ± SD is shown for nucleotide diversity (*p*), haplotype diversity (*H*_d_) and average number of pairwise differences (*k*). The *p* value and significance (*p* < 0.05) is shown for the neutrality tests. Tajima's *D* was calculated using *S* = 5, as gaps were excluded.

No. samples	Diversity statistics					Neutrality tests	
	No. haplotypes (*h*)	No. polymorphic sites (*S*)	Nucleotide diversity (*p*)	Haplotype diversity (*H*_d_)	Average no. pairwise differences (*k*)	Tajima's *D*	Fu's *F*
102	7	7	0.00195 (± 0.00130)	0.6329 (± 0.0334)	1.55 (± 0.93911)	−0.66145 (*p* = 0.291, N.S)	0.34125 (*p* = 0.617, N.S)

**TABLE 4:** Haplotype frequencies calculated in ARLEQUIN. Haplotype H2 is in bold as it was previously identified in Seddon et al. (2001) as E201/01; other haplotypes are newly identified.

Haplotype	Frequency	Relative frequency
H1	8	0.0784
H2	**52**	**0.51**
H3	33	0.324
H4	3	0.0294
H5	4	0.0392
H6	1	0.0098
H7	1	0.0098

**FIGURE 1:**
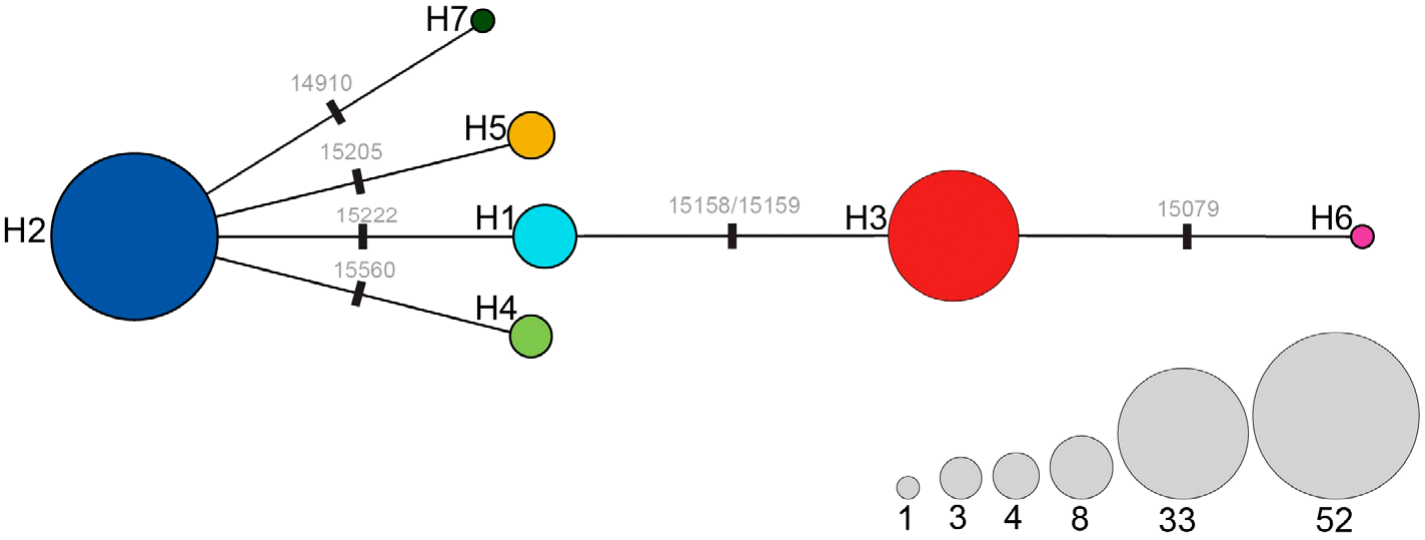
Median‐joining haplotype network of hedgehog samples in this study. Base changes and the position at which they occurred in the hedgehog mitochondrion genome (Krettek et al. 1995) are shown on the lines. Circle size is proportional to the number of individuals with each haplotype, scale shown in grey circles.

**FIGURE 2:**
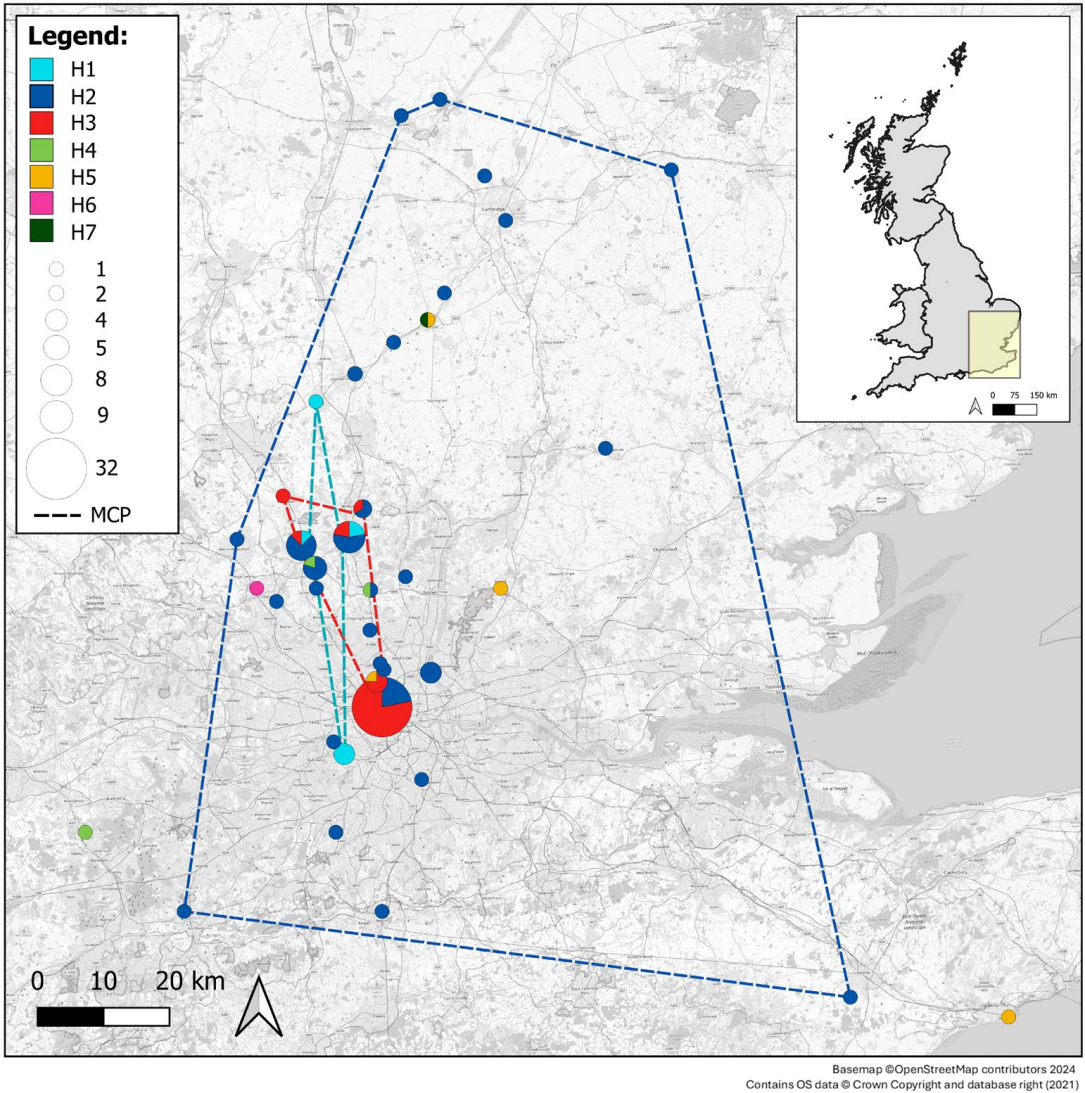
Map showing the distribution of hedgehog haplotype lineages. Pie charts represent the proportion of each haplotype at each location; the size indicates the number of samples. Minimum Convex Polygons (MCPs) are shown for H1, H2 and H3.

The median‐joining haplotype network reveals that four of the seven haplotypes in the study area radiate from the widespread H2 haplotype (Figure 1), which makes up 51% of all sequences, of which haplotype H1 has two further sequences branching linearly from it. H3 is the second most common haplotype, which arose due to a two‐base insertion in the control region, although this is likely due to bias in the sampling between locations, as the haplotype is common in the highly sampled Regent's Park location in central London (Figure 2).

### 4.2 Geographic Structure

Mapping haplotypes across the study area shows that H2 is widespread (Figure 2). This haplotype has previously been identified as being present throughout the UK (Seddon et al. 2001). No haplotypes were found to be unique to Greater London, with all haplotypes found within the city also present in surrounding counties. Furthermore, the less frequent haplotypes also showed broad distributions, with H5 for example being found on the south coast in Kent, as well as in Greater London, Essex and Cambridgeshire.

**FIGURE 3:**
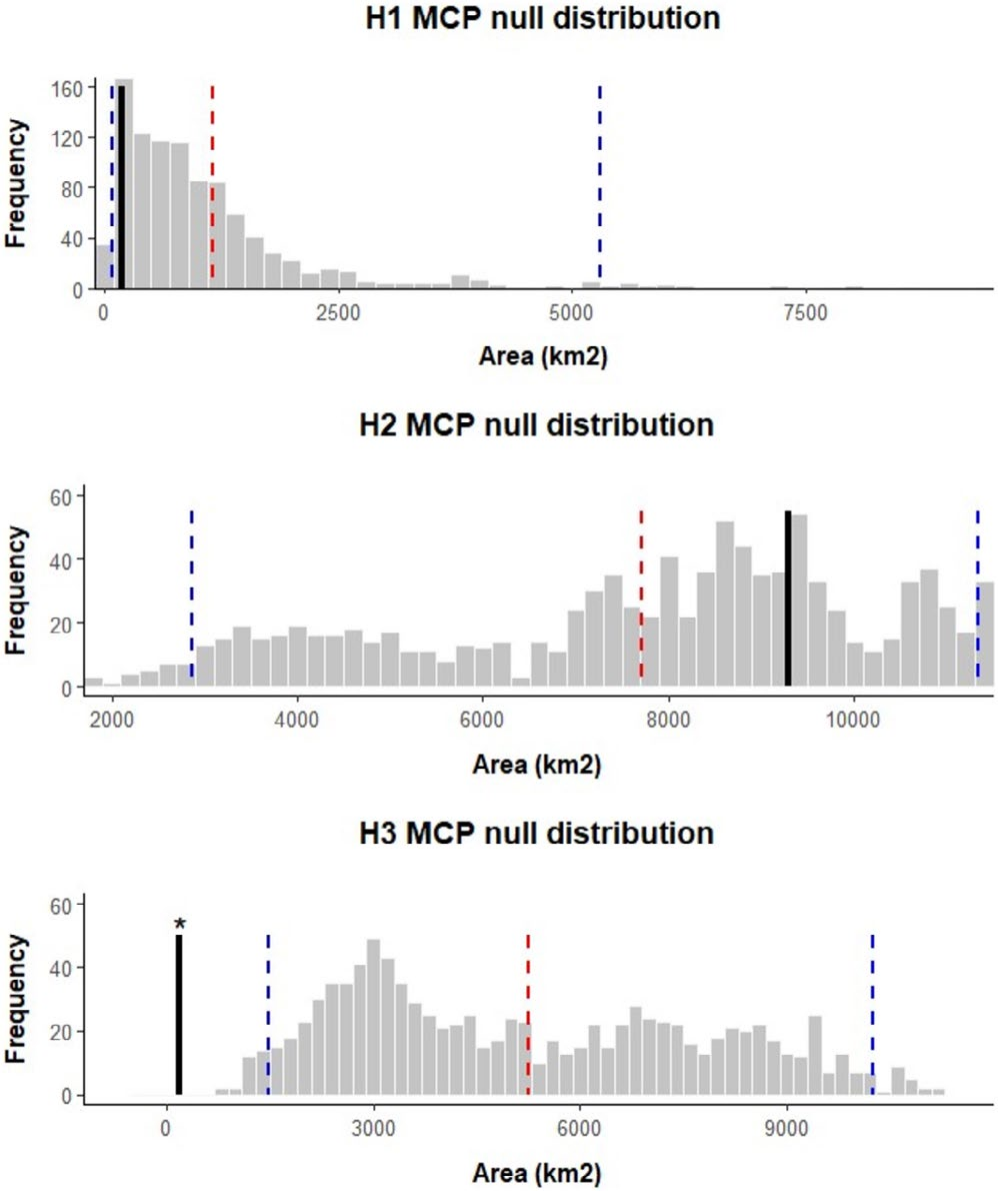
Histograms showing the expected distribution of Minimum Convex Polygon (MCP) areas of haplotypes H1 (8 detections at 4 sites), H2 (52 detections at 29 sites) and H3 (33 detections at 6 sites) under a null distribution after 1000 bootstrap replicates. The mean expected area (red), 95% confidence intervals (blue) and observed area (black) are shown as vertical lines. Significance (*p* < 0.05) is indicated by*. Only H3 has a spatial distribution significantly smaller distribution than expected.

**FIGURE 4:**
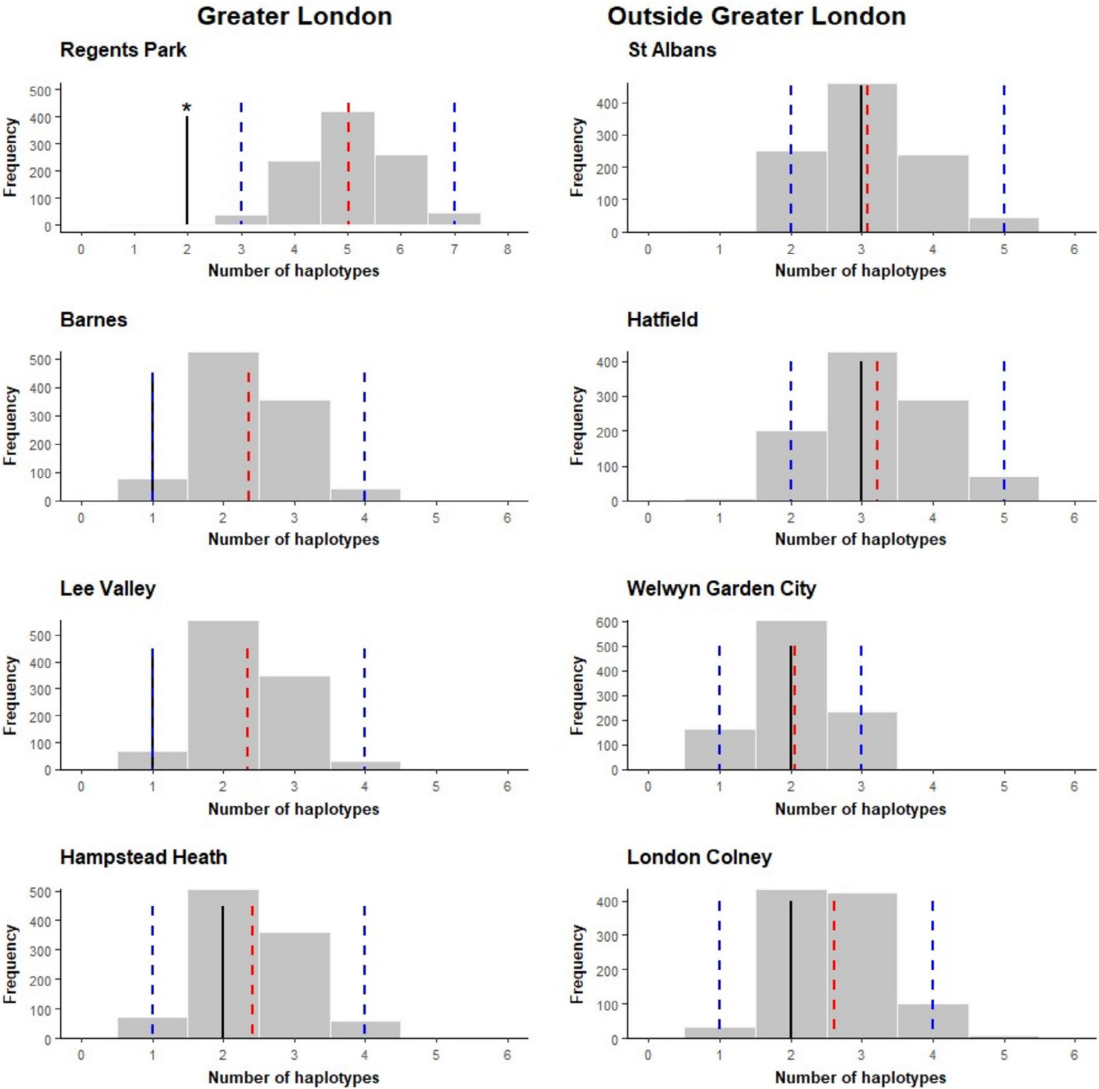
Histograms showing the expected distribution of haplotype diversity at each site with *n* ≥ 3 samples under a null distribution after 1000 bootstrap replicates. Sites within Greater London are on the left and sites outside Greater London on the right. The mean expected number of haplotypes (red), 95% confidence intervals (blue) and observed number of haplotypes (black) are shown as vertical lines. Significant results (*p* > 0.05) are indicated by*. Only Regent's Park contains significantly fewer haplotypes than expected, although Barnes and Lee Valley are close to significance as the observed and lower confidence interval lines overlap.

The spatial distributions of haplotypes H1, H2 and H3, which occurred in more than five samples, were assessed against the expectations of a null distribution to identify whether greater geographic clustering than random was present, indicative of restricted gene flow. The area over which haplotype H3 was found was significantly smaller (area: 190.69km^2^, 32 samples) than expected, whereas neither H1 nor H2 had a distribution significantly different from expected under the null distribution (Figure 3). Examination of the number of haplotypes present in sampling locations with multiple samples found that only one location (Regent's Park in central London) had significantly fewer haplotypes present than expected given the number of samples from that location (Figure 4), although two of the three other sites within Greater London also showed low haplotype diversity.

### 4.3 Comparison to Historical European Data

Sequences recovered by Seddon et al. (2001) were obtained from GenBank, and each haplotype was constructed using details from the paper. Overall, 65 *E. europaeus* and one *E. concolor* outgroup haplotypes were obtained. After alignment and trimming of sequences to match the haplotypes in the current study, three of these were combined into a single haplotype as nucleotide differences occurred in the trimmed regions; these were E1‐01/21 (Norway), E1‐01/22 (Sweden) and E1‐01/23 (Sweden). The E2‐01/01 and H2 haplotypes were also identical. A full list of haplotypes is provided in Table S2.

**FIGURE 5:**
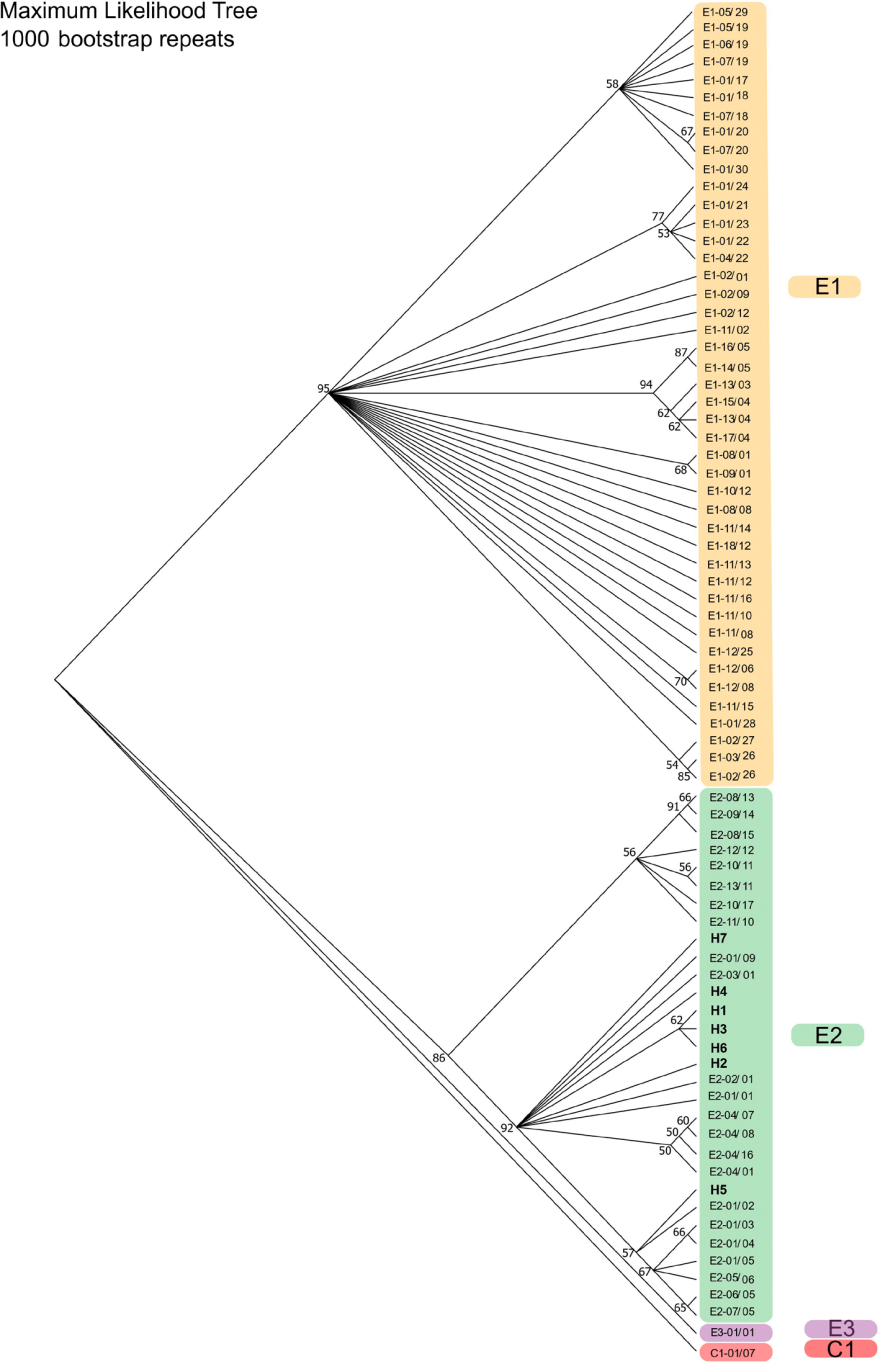
50% consensus maximum likelihood tree for western European hedgehog ( *E. europaeus* ) produced using HKY + G model of evolution. Nodes with < 50% support are polytomies, and unlabelled. Haplotypes are coloured to show the major E1, E2, E3, clades and southern white‐breasted hedgehog ( *E. concolor* ) outgroup C1.

The maximum likelihood tree constructed in MEGA11 failed to resolve relationships among haplotypes, with many polytomies and nodes with low support (Figure 5). The monophyly of the E2 lineage was supported in 95% of trees, and within this, six of the seven haplotypes from this study also grouped within a clade encompassing the UK, France, Jersey, the Netherlands, Switzerland and Germany. One haplotype, H5, grouped within a putative second group containing only samples from France and Jersey; however, the separation of this grouping was not strongly supported.

The Bayesian phylogeny showed similar topology to the ML tree, although constrained to a bifurcating tree structure (Figure S1). The tree identified monophyly of haplotypes H3, H6, H1, H7 and E2‐02/01, while H4 and H2 were closely related to E2‐03/01 and E2‐01/09, which occur in the Netherlands and France. However, these relationships had low posterior support, which may reflect the shallow differentiation between the haplotypes. As in the ML tree, H5 grouped within a separate second group within the E2 lineage found in France and Jersey, which was strongly supported (100% posterior probability).

In terms of molecular dating, the estimated divergence times of H3 and H6 (1130 years ago; 95% HPD 30–3775), H6 (2179 years ago; 95% HPD 181–6237), and H7 (5710 years ago; 95% HPD 2206–11,565) suggest that these may have originated after recolonisation to the UK, although with wide uncertainty. The divergence between the E2 and E1 clades was estimated at 135,188 years ago (95% HPD: 61,935—247,415), and E1 and E3 at 111,953 years ago (95% HPD 48.669–210,317), while the root divergence between *E. europaeus* and the outgroup *E. concolor* was dated at 375,500 years ago (95% HPD 162,997—694,964), but with large confidence intervals. These ages are later than those found in previous studies; for example, the divergence between western European hedgehogs and southern white‐breasted hedgehogs ( *E. concolor* ) has previously been estimated at about 1 million years ago (Mya) (0.83–1.19 Mya) (Eliášová et al. 2022) and 1.1 Mya (Bannikova et al. 2014).

The ancestral biogeographic range analysis conducted in Lagrange‐NG suggested a link between location and phylogenetic position (Figure S2). Haplotypes H1, H3, H6 and H7 are suggested to have originated in the UK, supporting the findings of the Bayesian phylogenetic analysis, whereas the ancestral location for H4, H5 and H2 could not be clearly identified.

**FIGURE 6:**
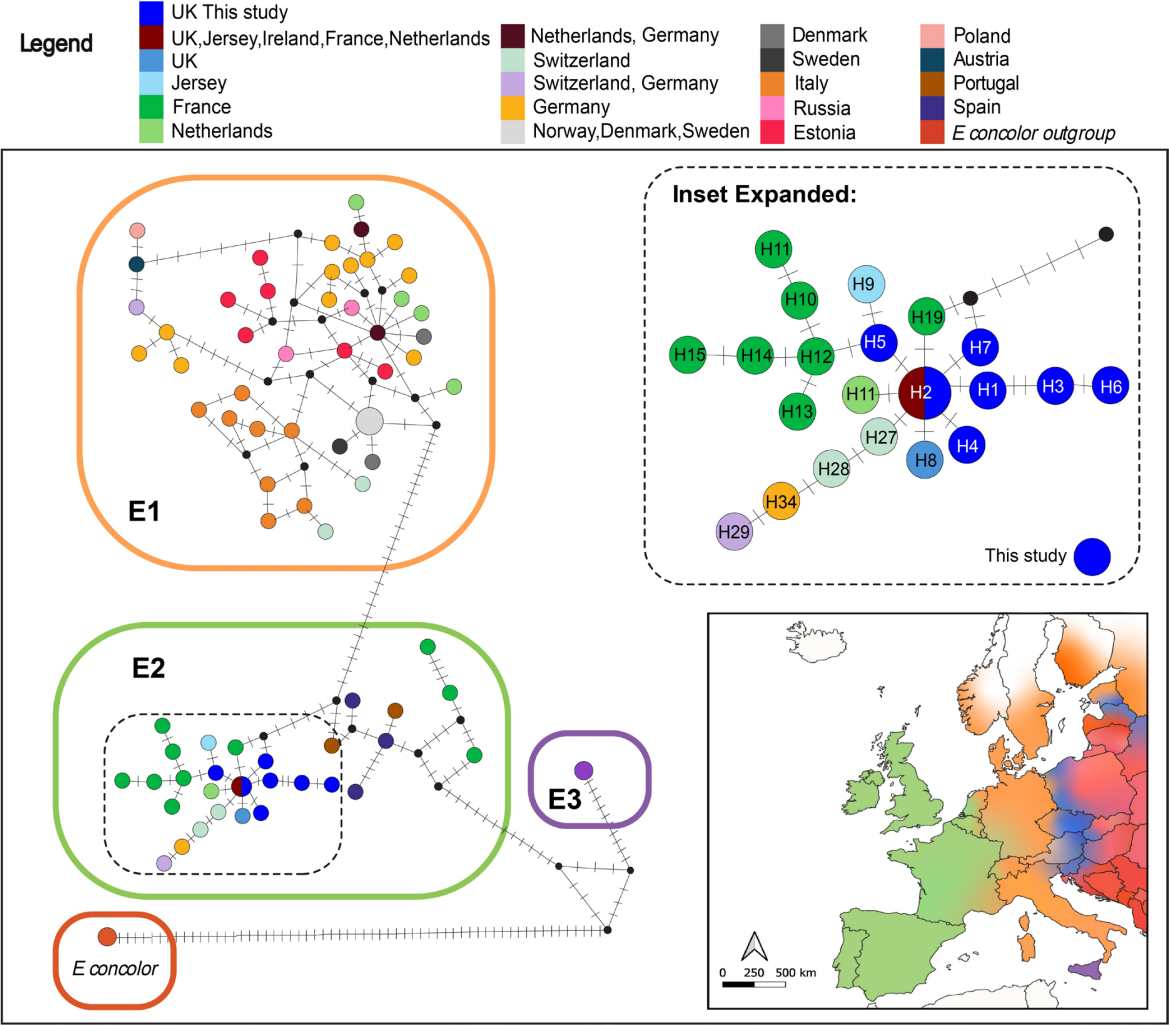
Median‐joining haplotype network of samples from this study and mainland Europe (Seddon et al. 2001). Haplotypes are coloured by country, with haplotypes from this study shown in blue. Clades are outlined in boxes (E1 orange, E2 green, E3 purple, outgroup *E. concolor* red). Top inset shows relationships of the labelled haplotypes from this study within the E2 clade (full haplotype names are in Table S2). Lower insert shows the distribution of clades, coloured as above, with hybrid zones with *E. concolor* shown in blue. Map boundaries were sourced from ‘rworldmap’ package (South 2011), clade and species distributions align with those of Seddon et al. (2001) and Eliášová et al. (2022).

Clear structuring of the hedgehog haplotypes is apparent in the median‐joining network (Figure 6) with multiple mutations separating the E1, E2 and E3 clades. Clade E3 shows a more complex structure, with several reticulations and inferred nodes. Within the E2 clade, there is a separation between moderately diverged haplotypes found in Spain, Portugal and France, and a second cluster of haplotypes which extends further northwards into France, Jersey, the Netherlands, Switzerland, Germany, Ireland and Great Britain. The second cluster (Figure 6, inset) shows a radiation of haplotypes with low sequence divergence from the widespread E2‐01/01 (H2 in this study) haplotype and includes the haplotypes found in this study. Interestingly, H7 forms a loop within the tree, linking to a haplotype found in France and an inferred unknown node, indicating uncertainty in the origin of this haplotype, while haplotype H5 is closely associated with haplotypes in France and Jersey.

Only eight of the previously published haplotypes across Europe, from Seddon et al. (2001), occurred in sufficient numbers for MCP analysis of their spatial distribution, all of which exhibited significant spatial clustering with smaller distributions than expected under a null distribution (map and plots shown in Figures S3 and S4). This was seen both when considering haplotypes from the E1 and E2 clades together and separately to account for their differing colonisation histories and contrasts with the limited evidence of geographic structuring seen in the MCP analysis of haplotypes within the UK samples in this study.

## 5 Discussion

This study presents a detailed investigation into the phylogeography of hedgehogs in Greater London, east and south‐east England using partial mitochondrial *Cytb* and *CR* gene regions and relates these findings to the broader phylogeographic structure of hedgehogs across Europe. We find higher mitochondrial diversity than previously found in the UK (Seddon et al. 2001) with seven haplotypes identified in the study region, of which six are novel and may be distinct to the UK. Examination of the spatial structuring of haplotypes found limited evidence of geographic substructure in the study region, in contrast to significant spatial clustering in haplotypes at the broader European scale. However, there was evidence of low haplotype diversity in some areas of central London, suggesting potential genetic isolation in the urban environment.

Previous studies of hedgehog phylogeography have included relatively few samples from the UK. Three individuals from the east of England were found to have a single *Cytb* haplotype (Santucci et al. 1998), while further investigation of 28 individuals, including an additional *CR* sequence, also found a single widespread haplotype in most sample locations and a second haplotype restricted to a single location on the south coast (Seddon et al. 2001). These results are consistent with the loss of genetic diversity with increasing latitude due to serial population bottlenecks during postglacial expansion (Hewitt 1999). This pattern has also been seen in European badgers ( *Meles meles* ), for example, in which individuals from Scandinavia, Britain and Ireland have been found to be less genetically variable than more southern populations (Frantz et al. 2014).

In the present study, we increase the sample size considerably to include 102 samples from Greater London and surrounding counties, and find higher diversity with six newly identified haplotypes. It is possible that these novel haplotypes originated within the UK, as the divergence among them is small. Bayesian molecular dating analysis supported recent divergence of several of these haplotypes, and Lagrange‐NG analysis also suggested geographic structuring within the phylogeny, with the ancestral nodes of five haplotypes (H1, H3, H6, H7 and E2‐02/01) inferred to have originated within the UK. However, as the differences between the haplotypes are small, it is challenging to assess this certainty, and a larger data set with more variable sites would be required to obtain a clearer understanding. A similar pattern of shallow differentiation of mitochondrial lineages in the UK has also been observed in the common dormouse ( *Muscardinus avellanarius* ), with haplotypes separated by a single mutation thought to have originated after the UK separated from mainland Europe (Combe et al. 2016). Furthermore, similar patterns of shallow lineage radiations are also seen in hedgehogs in France and the Netherlands (Figure 6), with haplotypes generally separated by only one mutation.

Alternatively, it is also possible that these haplotypes were present in the Iberian Peninsula prior to the northward recolonisation of hedgehogs, as it has been suggested that most haplotypes were likely to have been present in refugia due to the recentness of hedgehog postglacial expansion (Seddon et al. 2001). Haplotype H1, for example, has two further derivative haplotypes and may therefore predate the isolation of the UK from mainland Europe, but further sampling in mainland Europe would be required to identify whether it is present there. Interestingly, recent analyses of hedgehog phylogeography within the Iberian Peninsula do not appear to support this, finding that the northward expansion of hedgehogs originates from the less diverse of two clades present in the region (Sanz et al. 2021). There, only the E201 *Cytb* haplotype was present in Iberia, with other *Cytb* haplotypes across mainland Europe differing from this by 1 or 2 mutations, similar to haplotypes H6 and H7 reported in the UK in the present study. However, it is important to note that the control region, which is more variable, was not included in the Iberian Peninsula study by Sanz et al. (2021).

Phylogeographic substructure within regions can indicate recent constraint to gene flow, for example, haplotypes that are common in a small area but absent from the wider landscape may indicate geographic barriers to gene flow (Avise 1992). In this study, only limited evidence of spatial geographic clustering was identified in and around Greater London. None of the haplotypes observed more than once were considered ‘private’, defined as being found in one or adjacent locations (Avise 1992). Investigation of spatial clustering in the three most frequent haplotypes in the data set found only one, H3, to be significantly spatially clustered, primarily seen in central and north London and in Hertfordshire. However, this result should be treated with caution due to the strong bias in sampling between the central London Regent's Park site, where this haplotype is common, and other sample sites in the data set. Haplotypes H1 and H3 showed no significant difference in distribution to that expected under a null distribution.

In contrast, investigation of the spatial distribution of haplotypes identified across mainland Europe (Seddon et al. 2001) found significantly smaller distributions than expected in all eight haplotypes with sufficient records to be investigated. This may reflect the generation of new haplotypes that then become locally common occurring across mainland Europe, similar to the identification of a high diversity of haplotypes within the UK, rather than the widespread distribution of older refugial haplotypes. This is also concordant with the findings of the Bayesian molecular dating analysis and model of geographic range evolution in Lagrange‐NG. However, it is important to note that only a very limited number of haplotypes (eight from 65) were included in this analysis, and sampling per location was sparse and may not reflect the true distributions of the haplotypes in Europe. In this way, further comparative analysis of hedgehogs from the UK and from across mainland Europe, with denser sampling and additional genetic markers, would provide useful insights into local haplotype divergence in hedgehogs and further understanding regarding potential divergence between the UK and mainland Europe.

Distinct substructure in hedgehog lineages at smaller landscape scales has been identified in previous studies; for example, three subpopulations separated by major rivers were identified in the Czech Republic (Bolfikova and Hulva 2012). In Finland, investigation of phylogeography and population structure of hedgehogs in an urban landscape identified a spatially biased distribution of mitochondrial haplotypes, with two of the four identified haplotypes restricted to the urban areas of Helsinki, Espoo and Vanta, whereas a third was found only outside of this region, and the fourth was widespread (Osaka et al. 2022). In contrast, all haplotypes identified within Greater London in this study were also observed outside of the city, indicating either multiple colonisation events from surrounding areas (Menke et al. 2010), or persistence of populations present before urbanisation occurred. In addition, the Thames River, which bisects Greater London, does not appear to be a major barrier within the region, although it is important to note that this study included few individuals from south of the Thames, and further sampling would be needed to explore this in greater detail.

In addition to spatial clustering of haplotypes, variation in haplotype diversity may also be informative regarding population structure and gene flow. Absence of haplotype diversity may indicate population bottlenecks, as haplotypes can be lost when population size is reduced, or lack of gene flow preventing the introduction of new mitochondrial lineages. Interestingly, the frequency with which haplotypes were observed at locations did show some local differences, with H3 accounting for over three quarters of recovered haplotypes in The Regent's Park and Hampstead Heath within Greater London, and H1 being the only haplotype seen in Barnes. In counties north of Greater London, the widespread H2 haplotype was the most frequently identified. Comparison of observed haplotype diversity at sites compared to that expected under a null distribution identified that, for the four sampling locations examined outside of Greater London, the number of haplotypes found was close to the mean expected value. In contrast, within Greater London, the number of haplotypes recovered was close to the expected mean in just one site (Hampstead Heath, 2 haplotypes), at the lower confidence limits at two sites (Barnes and Lee Valley, with 1 haplotype), and significantly lower than expected at one site (Regent's Park, with 2 haplotypes). This may be indicative of potential small population size or past bottlenecks, leading to loss of haplotype diversity, and limited gene flow enabling local haplotype frequencies to diverge from that seen in the wider landscape. The Regent's Park population, for example, is known to be small and to have undergone severe population fluctuations in recent years (*pers comm*. The Royal Parks), while the population in Barnes is surrounded on three sides by a bend in the river Thames.

Similar patterns of mitochondrial divergence in urban areas have also been seen in other species. The Jerusalem cricket (*Stenopelmatus* n. sp) in Los Angeles, California, for example, was found to have high mtDNA divergence among populations in urban habitat fragments (Vandergast et al. 2009). In West Tokyo, Japan, isolated urban populations of large Japanese field mice ( *Apodemus speciosus* ) contained only 1–2 haplotypes, whereas 4–8 haplotypes were found in a contiguous landscape along the Tama river (Hirota et al. 2004). These results suggest that the urban landscape may play a role in isolating hedgehog populations and generate substructure through fragmentation. However, it is important to note that per site sampling intensity in this study is low at most sites, and further sampling would be required to identify whether the findings of our study accurately represent the true haplotype frequencies in the wider or national population (Avise 1992).

Hedgehogs are generally philopatric, with home ranges of 10–12 ha in females and 35–40 ha in males (P. A. Morris 1988). Dispersal events up to 15 km have been recorded (P. Morris 2018), but it is considered that dispersal over 4 km is uncommon (Doncaster et al. 2001). Therefore, it would be expected for hedgehogs to show some regional substructuring, particularly due to natural barriers such as less suitable upland regions or large rivers. It is possible that the area and sample size over which the study was conducted were too small to detect structuring, and therefore further investigation of hedgehog phylogeography across the UK would be valuable to explore whether broader patterns exist.

In several small mammals, UK‐wide phylogeographic patterns have been found with a ‘Celtic Fringe’ showing distinct mitochondrial clusters in Wales, Scotland and Cornwall as a result of a two‐stage colonisation process (Searle et al. 2009). It is probable that further mitochondrial diversity exists in the UK outside of the study region reported here. Analysis of the origins of hedgehogs in New Zealand, for example, which originated from the UK in the late 19th to early 20th Century, found five control region haplotypes, two of which the authors also found in samples from the UK (Bolfikova et al. 2013). These were not found to be identical to any of the haplotypes reported here and present further evidence of higher mitochondrial diversity being present in the UK than previously recognised. Furthermore, with sampling of longer gene regions, it is possible that further diversity will be identified, as three unique haplotypes from mainland Europe were condensed to a single haplotype when the sequences were shortened for comparison to the sequences in this study from the UK.

It is also important to consider the potential influence of human‐mediated movement of hedgehogs on the distribution of haplotypes. Hedgehogs have been introduced to new locations through human‐mediated movement, including the Uists, Scotland (Jackson 2006), New Zealand (Bolfikova et al. 2013) and Pianosa Island, Italy (Iannucci et al. 2019). In addition, hedgehogs are the most common wildlife species taken into wildlife hospitals in the UK (Molony et al. 2006; Mullineaux and Pawson 2023), and it was estimated that 40,000–56,000 were admitted to wildlife rehabilitators in 2016 (Bearman‐Brown and Baker 2022). While best practice is for rescued hedgehogs to be released where they were found whenever possible (Guidance for Releasing Hedgehogs that have been rehabilitated—A collaborative View [online]), translocations may be conducted on release from rehabilitation centres to avoid re‐exposure to threats where they were originally found (Molony et al. 2006), which could have an impact on the population genetic structure.

Comparing the relationship of haplotypes in this study to the rest of Europe, haplotype H5 was found to be closely related to haplotypes from Jersey and France (H9 and H12 in Figure 6, inset box 1, full haplotype names in Table S2) in the median‐joining network, ML and Bayesian phylogenetic trees. Several further haplotypes derive from the French haplotype H12 in the haplotype network and may indicate that haplotype H5 originated in France and moved to the UK through Doggerland prior to the formation of the English Channel. More intense sampling in France would be useful to identify whether further close association with haplotypes in the UK is present. In addition, a loop within the network was identified between H7 and H2 from this study, and haplotype H19 from France and an unknown ancestral haplotype. This loop may be due to conflict arising between the characters due to convergent events, in which the same position has mutated separately (Morrison 2005), or due to sampling error, as H7 was only observed once in the data set.

## 6 Conclusion

Overall, maintaining genetic diversity and natural genetic structuring in mammals is desirable in many conservation efforts, and may be at risk of being disrupted through actions such as translocation. In this study, new mitochondrial haplotype diversity is identified in the east and south‐east of England, including Greater London and surrounding counties, and potentially suggests unique diversification of hedgehog mitochondrial lineages within the UK. However, there was limited evidence of geographic structure in the study area, in contrast to high levels of spatial clustering in haplotypes across mainland Europe. Therefore, within the study region, disruption of local structure in mitochondrial genetic diversity may not be a major concern for hedgehogs, potentially due to the already high levels of human‐mediated movement of the species within the UK. Further investigation into the genetic consequences of rehabilitation in hedgehog populations would be valuable to understand these impacts. Despite having overall high haplotype diversity in Greater London, we find some evidence of local variation in haplotype frequencies and an absence of haplotype diversity within urban sites in Greater London, possibly as a result of local population bottlenecks and limited dispersal in the urban environment. While this requires further investigation, which is currently being undertaken using a more comprehensive data set of nuclear single nucleotide polymorphism (SNP) markers to examine population and landscape genetic patterns (Turner et al. 2021) it may have implications for targeting of conservation efforts in urban areas to increase gene flow in these environments. In future, extending the investigation of detailed hedgehog mitochondrial phylogeography across the UK will be important to identify the presence of further haplotype diversity and broader scale phylogeographic structure.

## Author Contributions

**Jessica Turner:** conceptualization (equal), data curation (lead), formal analysis (lead), investigation (lead), resources (lead), writing – original draft (lead), writing – review and editing (equal). **Chris Carbone:** conceptualization (equal), investigation (supporting), resources (supporting), supervision (equal), writing – review and editing (equal). **Becki Lawson:** data curation (supporting), resources (supporting), writing – review and editing (equal). **Katharina Seilern‐Macpherson:** data curation (supporting), resources (supporting), writing – review and editing (equal). **Chris Faulkes:** conceptualization (equal), investigation (supporting), resources (supporting), supervision (equal), writing – review and editing (equal).

## Conflicts of Interest

The authors declare no conflicts of interest.

## Supporting information


Appendix S1.

## Data Availability

New nucleotide sequences from this study are available from GenBank (Accession numbers: PQ772616‐PQ772621). The haplotype dataset generated and analysed in this study is available at https://doi.org/10.5061/dryad.6wwpzgn86. Previously published data used in this study is already available from their original sources.
