# Supplementary material for: Fine Scale Phylogeography of Urban Western European Hedgehog Erinaceus europaeus in South‐East England
Source: Ecol Evol. 2025 Jul 17;15(7):e71729. doi: 10.1002/ece3.71729 (PMC12271519; doi:10.1002/ece3.71729)
Supplement: Supplementary file 1 — Appendix S1. [file ECE3-15-e71729-s001.docx]

**Appendices**

|  | Diversity statistics | | | | | Neutrality Tests | |
| --- | --- | --- | --- | --- | --- | --- | --- |
| No. samples | No. haplotypes (h) | No. polymorphic sites (S) | Nucleotide diversity (p) | Haplotype diversity (Hd) | Average no. pairwise differences (k) | Tajima’s D | Fu’s F |
| 102 | 6 | 5 | 0.00087 | 0.582 (+/- 0.027) | 0.662 | -0.66145  (P = 0.291, N.S) | -1.635  (P = 0.617, N.S) |

Table S1: DnaSP Diversity statistics and neutrality tests carried out in Dnasp V6, gaps are excluded. Mean +/- SD are shown for nucleotide diversity, haplotype diversity and pairwise differences, and the significance (P <0.05) is shown for the neutrality test statistics.

| Haplotype | Previous naming | Country |
| --- | --- | --- |
| H1 | - | THIS STUDY |
| H2 | H2/  E201/01 | THIS STUDY  UK, JERSEY, IRELAND, FRANCE, NETHERLANDS |
| H3 | - | THIS STUDY |
| H4 | - | THIS STUDY |
| H5 | - | THIS STUDY |
| H6 | - | THIS STUDY |
| H7 | - | THIS STUDY |
| H8 | E2-02/01 | UK |
| H9 | E2-01/02 | JERSEY |
| H10 | E2-01/03 | FRANCE |
| H11 | E2-01/04 | FRANCE |
| H12 | E2-01/05 | FRANCE |
| H13 | E2-05/06 | FRANCE |
| H14 | E2-06/05 | FRANCE |
| H15 | E2-07/05 | FRANCE |
| H16 | E2-08/15 | FRANCE |
| H17 | E2-08/13 | FRANCE |
| H18 | E2-09/14 | FRANCE |
| H19 | E2-01/09 | FRANCE |
| H20 | E2-03/01 | NETHERLANDS |
| H21 | E1-11/16 | NETHERLANDS |
| H22 | E1-11/12 | NETHERLANDS, GERMANY |
| H23 | E1-11/14 | NETHERLANDS |
| H24 | E1-12/06 | NETHERLANDS |
| H25 | E1-12/08 | NETHERLANDS, GERMANY |
| H26 | E1-12/25 | NETHERLANDS |
| H27 | E2-04/01 | SWITZERLAND |
| H28 | E2-04/16 | SWITZERLAND |
| H29 | E2-04/07 | SWITZERLAND, GERMANY |
| H30 | E1-01/30 | SWITZERLAND |
| H31 | E1-05/29 | SWITZERLAND |
| H32 | E1-13/03 | SWITZERLAND, GERMANY |
| H33 | E1-13/04 | GERMANY |
| H34 | E2-04/08 | GERMANY |
| H35 | E1-11/10 | GERMANY |
| H36 | E1-08/01 | GERMANY |
| H37 | E1-09/01 | GERMANY |
| H38 | E1-10/12 | GERMANY |
| H39 | E1-11/02 | GERMANY |
| H40 | E1-11/08 | GERMANY |
| H41 | E1-15/04 | GERMANY |
| H42 | E1-08/08 | GERMANY |
| H43 | E1-17/04 | GERMANY |
| H44 | E1-18/12 | GERMANY |
| H45 | E1-01/21  E1-0/23  E1-01/22 | NORWAY, DENMARK  SWEDEN  SWEDEN |
| H46 | E1-01/24 | DENMARK |
| H47 | E1-04/22 | SWEDEN |
| H48 | E1-11/13 | DENMARK |
| H49 | E1-05/19 | ITALY |
| H50 | E1-06/19 | ITALY |
| H51 | E1-01/18 | ITALY |
| H52 | E1-01/20 | ITALY |
| H53 | E1-07/19 | ITALY |
| H54 | E1-07/20 | ITALY |
| H55 | E1-07/18 | ITALY |
| H56 | E1-01/17 | ITALY |
| H57 | E1-11/15 | RUSSIA |
| H58 | E1-02/01 | RUSSIA |
| H59 | E1-02/09 | ESTONIA |
| H60 | E1-02/12 | ESTONIA |
| H61 | E1-03/26 | ESTONIA |
| H62 | E1-02/26 | ESTONIA |
| H63 | E1-02/27 | ESTONIA |
| H64 | E1-01/28 | ESTONIA |
| H65 | E1-16/05 | POLAND |
| H66 | E1-14/05 | AUSTRIA |
| H67 | E2-10/17 | PORTUGAL |
| H68 | E2-10/11 | SPAIN |
| H69 | E2-13/11 | PORTUGAL |
| H70 | E2-12/12 | SPAIN |
| H71 | E2-11/10 | SPAIN |
| H72 | E3-01/01 | ITALY |
| H73 | C1-01/07 | ITALY |

Table S2: List of all haplotypes showing haplotype name generated in this study (“Haplotype”), the Seddon *et al.* 2001 designation (“Previous naming”), and country of occurrence.


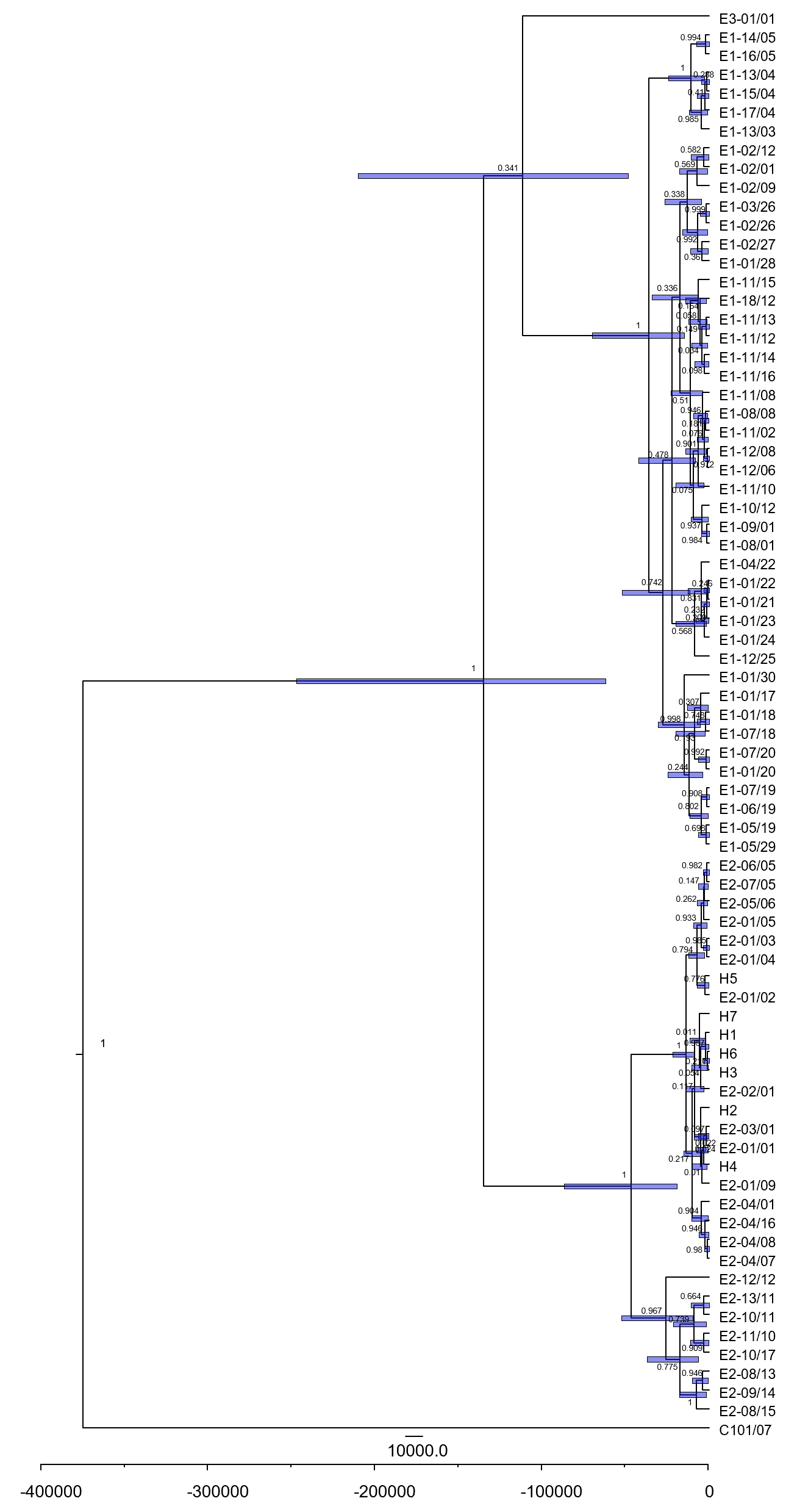


Figure S1: Maximum clade credibility tree for western European hedgehog (*E. europaeus)* inferred using BEAST with a strict molecular clock and calibrated using recolonisation of haplotypes found in the UK after the Last Glacial Maximum (mean: 13,750 years ago, CI: 10,700 – 17,000 years ago). Blue bars spanning the nodes correspond to ages for the upper and lower bound of the 95% highest posterior density (HPD) intervals. The 95% HPD for the root of the tree is not shown. Numbers at nodes refer to the posterior probabilities in support of that node (1 = 100%).


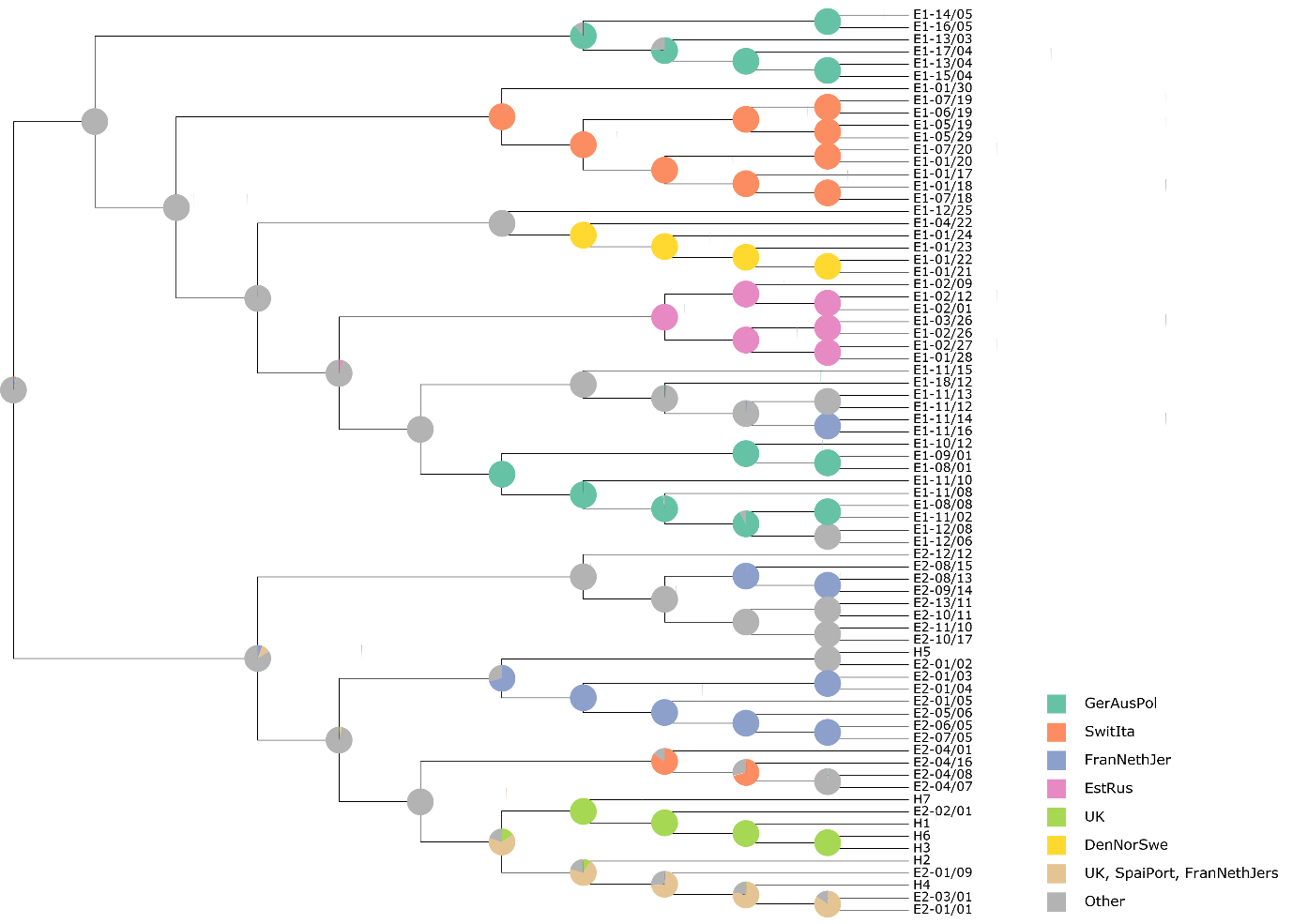


Figure S2: Ancestral biogeography of western European hedgehog (*E. europaeus*) inferred from Lagrange-NG analysis. Pie charts at interior nodes represent the likelihood weight ratio of the top distributions. Legend codes: GerAusPol = Germany, Austria, Poland. SwiIta = Switzerland, Italy. FranNethJers = France, Netherlands, Jersey. EstRus = Estonia, Russia, UK = UK, SpaiPort = Spain, Portugal, DenNorSwe = Denmark, Norway, Sweden.


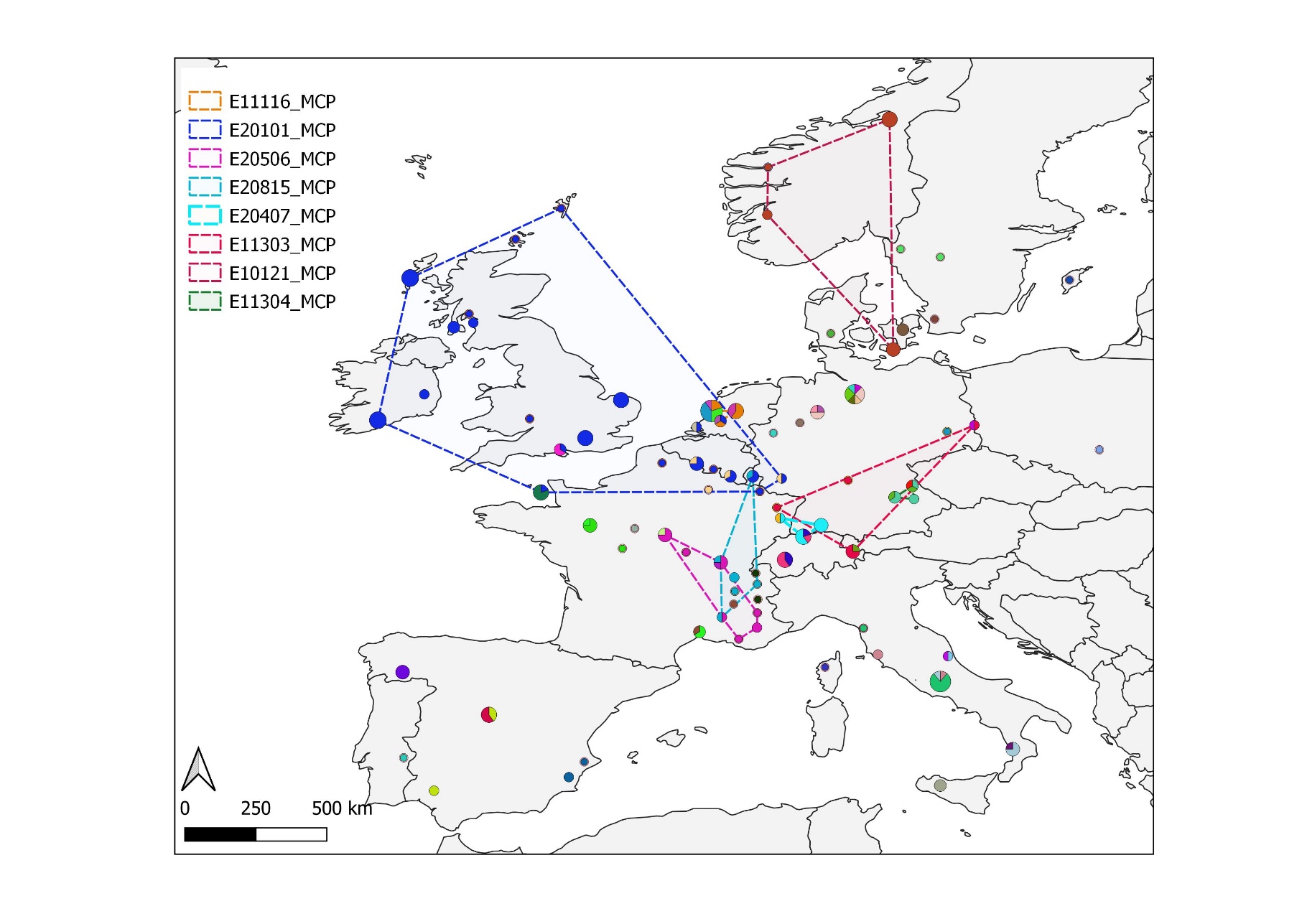


Figure S3: Map showing distribution of haplotypes found in Seddon *et al.* (2001) Pie charts represent the proportion of each haplotype at each location and size indicates the number of samples. Minimum Convex Polygon (MCP) areas are shown for eight haplotypes with more than five occurrences in the dataset (4 from E1, 4 from E2 clades). Background map is from rworldmap (South, 2011).


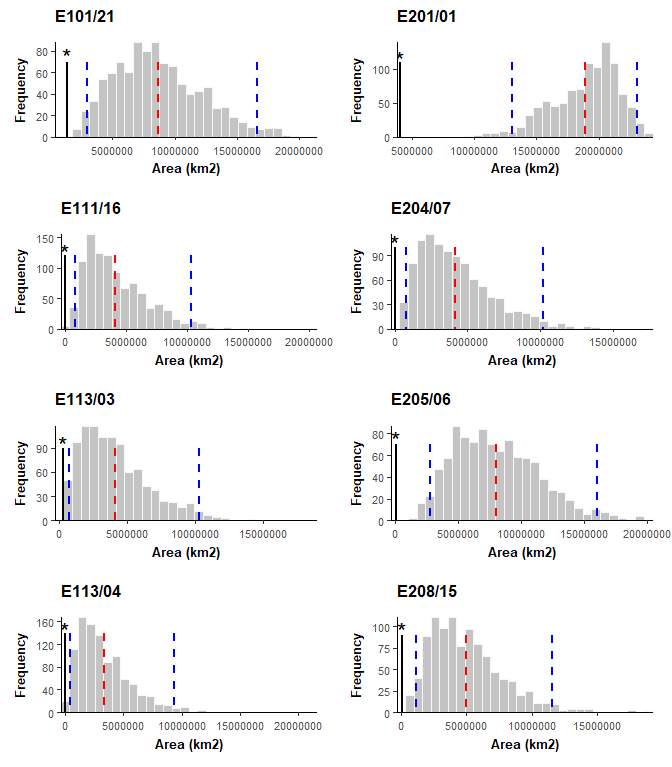


Figure S4: Histograms showing the expected distribution of Minimum Convex Polygon (MCP) areas of eight European haplotypes from Seddon *et al.* (2001) under a null distribution after 1000 bootstrap replicates. The mean expected area (red), 95% confidence intervals (blue), and observed area (black) are shown as vertical lines. Significant results (p >0.05) are indicated by *.
